# Keratin Biomaterials in Skin Wound Healing, an Old Player in Modern Medicine: A Mini Review

**DOI:** 10.3390/pharmaceutics13122029

**Published:** 2021-11-28

**Authors:** Marek Konop, Mateusz Rybka, Adrian Drapała

**Affiliations:** Laboratory of Center for Preclinical Research, Department of Experimental Physiology and Pathophysiology, Medical University of Warsaw, 02-106 Warsaw, Poland; mateuszrybka@mp.pl (M.R.); adrapala@wum.edu.pl (A.D.)

**Keywords:** keratin, keratin wound dressing, skin wound healing, tissue regeneration, regenerative medicine

## Abstract

Impaired wound healing is a major medical problem. To solve it, researchers around the world have turned their attention to the use of tissue-engineered products to aid in skin regeneration in case of acute and chronic wounds. One of the primary goals of tissue engineering and regenerative medicine is to develop a matrix or scaffold system that mimics the structure and function of native tissue. Keratin biomaterials derived from wool, hair, and bristle have been the subjects of active research in the context of tissue regeneration for over a decade. Keratin derivatives, which can be either soluble or insoluble, are utilized as wound dressings since keratins are dynamically up-regulated and needed in skin wound healing. Tissue biocompatibility, biodegradability, mechanical durability, and natural abundance are only a few of the keratin biomaterials’ properties, making them excellent wound dressing materials to treat acute and chronic wounds. Several experimental and pre-clinical studies described the beneficial effects of the keratin-based wound dressing in faster wound healing. This review focuses exclusively on the biomedical application of a different type of keratin biomaterials as a wound dressing in pre-clinical and clinical conditions.

## 1. Introduction

Keratin biomaterials have been in use for decades, yet they are seemingly new to the field. Keratin is a global class of biological material, which represents a group of cysteine-rich filament-forming proteins [1,2]. It is the major component of hair, wool, hooves, nails, feathers, and horns are one of the most abundant and underexploited protein sources [3]. Their complex hierarchical structure, such as filament-matrix composition at nano-level and polypeptide chains, create a robust wall for protection against heat stress, pathogen invasions (particularly through the skin), and mechanical damage [1,2,4]. Keratins and keratinous materials are subdivided into two different classes of secondary structures of protein: alpha (*α*)-keratin and beta (*β*)-keratin [4]. Moreover, keratins are the protein that forms a shielding layer for the epidermal appendages and, thereby, imparting an important role in protection. Keratin genes account for most of the intermediate filament genes in the human genome, making up the two largest sequence homology groups: keratins type I, also called acidic keratins; and keratins type II, called neutral-basic keratins [1,2,4].

Different keratins play different roles in the wound healing process, so it should be noted that their expression varies, depending on the stage of the wound healing process. In addition, various extraction methods allow the creation of various forms of keratin biomaterials, including films, sponges, gels hydrogels, and scaffolds [5]. Keratin materials have been extensively used in tissue engineering and regenerative medicine owing to their biological function, structural support, excellent biocompatibility, and favorable biodegradability characteristics. Accordingly, these properties allow scientists to create a new type of wound dressing that can enhance the healing process especially in chronic non-healing wounds [6,7,8,9,10]. They can be cast as sponges, films, and hydrogels for various biomedical applications [11]. In this mini review, we focused on the biomedical application of a different type of keratin biomaterials as a wound dressing in pre-clinical and clinical settings.

## 2. Features of the Ideal Wound Dressing

Impaired wound healing is a major medical problem in the modern world. Scientists around the world try to create an ideal wound dressing that could accelerate the recovery of non-healing wounds. It should be mentioned that there is no single dressing that could be used for all types of wounds. However, during tissue recovery of a single wound, different types of wound dressings could be applied. In 1989, Turner described the general criteria which an ideal dressing should possess [12,13]. The dressing in contact with the wound must provide a moist environment, while absorbing wound fluids [12,14,15]. It should maintain appropriate tissue temperature to improve the blood flow to the wound [16]. It should be biocompatible, biodegradable [7,10,17], semi-permeable to water, and oxygen [18]. Moreover, it should promote tissue remodeling and should not provoke tissue immune responses [16]. Furthermore, changing the dressing should be painless and should not damage the newly formed tissue. It is important for the dressing to be also cost-effective [12]. In the last few years, several new dressings have been developed to improve tissue regeneration. They are indicated for specific types of chronic wounds based on a wound condition, such as being dry or exuding, superficial or deep, and clean or infected [19]. Recent progress in material sciences has been dedicated to the possibility of adding drugs, growth factors, stem cells, or silver nanoparticles into the wound dressing to improve it [10,20,21,22,23]. These active components should dynamically interact with the surrounding tissue and improve healing either by helping the removal of necrotic tissues or preventing secondary infections [12,24]. It has to be pointed out that wound dressings containing antibiotics are valuable in the management of local (where the highest concentrations of applied antibiotics are required), but not systemic, infections. Nevertheless, in some cases, high amounts of antibiotics or nanoparticles can lead to systemic toxicity and impaired wound healing [12,23,25].

## 3. Structure and Function of the Skin

**Figure 1:**
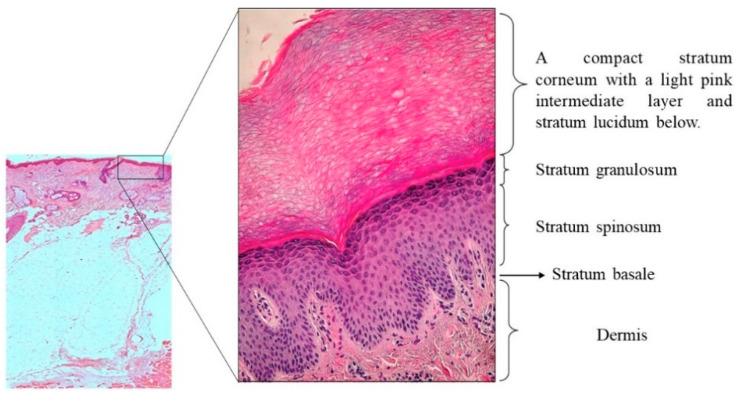
Structure of the skin end epidermis (courtesy of J. Czuwara, MD, Ph.D.). Magnification 200× (online, in color; black and white, in print).

Skin is the biggest human complex and multifunctional organ; see Figure 1. It carries out many vital functions, including protection against external physical, chemical, and biological agents. 

By the same token, it prevents excess water loss from the body and participates in the thermoregulation process. From a histological point of view, it consists of two layers, the epidermis, and dermis, separated by a basement membrane zone [26,27] and subcutaneous tissues.

### 3.1. Epidermis

The epidermis is the outer layer of the skin and constitutes a barrier that denies the entry of pathogens and foreign bodies to the body. By the way, it protects the body from DNA damage caused by ultraviolet (UV) radiation and retains water [28,29,30]. In 95%, it consists of keratinocytes, followed by melanocytes, Langerhans cells, and Merkle cells. These cells represent 5% of the total cells existing in this layer. A keratinocyte is a cell that manufactures and stores the protein called keratin. The epidermis consists of four layers: stratum basale, stratum spinosum, stratum granulosum, and stratum corneum, depending on the state of keratinocyte differentiation [27,31]. The epidermis is a continually renewing skin layer and gives rise to derivative structures, such as hair and nails. The full regeneration process takes about 40–48 days [27,28,29,31].

### 3.2. Basement Membrane Zone (BMZ)

Basement membrane zone (BMZ) is histologically defined by a 0.5–1.0 mm-thick band-like structure that separated the epidermis from the dermis [32]. It is a complex, precisely organized, and dynamic collection of proteins that provide structure and regulate cell adhesion, differentiation, motility, signal transmission, and membrane permeability. This skin region consists of an intricate network of macromolecules that link the keratin intermediate filaments of basal keratinocytes with collagen fibers in the superficial dermis [33]. The main function of the proteins and glycoproteins within the BMZ is to provide adhesion between the epidermis and the dermis [34,35]. 

**Figure 2:**
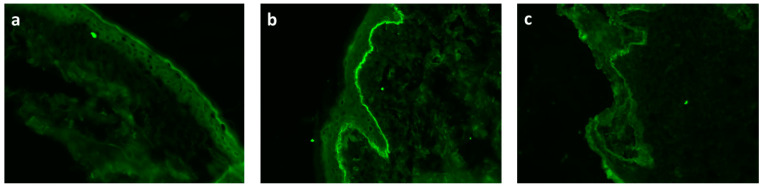
Direct immunofluorescence of skin biopsy in patients with bullous pemphigoid disease (**a**) control skin, (**b**) positive staining for BMZ in the skin, (**c**) positive staining for BMZ in mucosa membrane). Magnification 200× (private photography) (online, in color; black and white, in print).

Any abnormalities or defects observed in these molecules result in skin blistering diseases, such as pemphigoid and pemphigus diseases. The pemphigoid group is characterized by autoantibody-mediated inflammation at the dermal–epidermal junction (DEJ) with autoantibodies against components of the BMZ (Figure 2) [36,37]. The pemphigus group is characterized by the loss of connection between keratinocytes and cells of the epidermal spinosum (stratum spinosum) through the loss of intercellular bridges [38]. 

**Figure 3:**
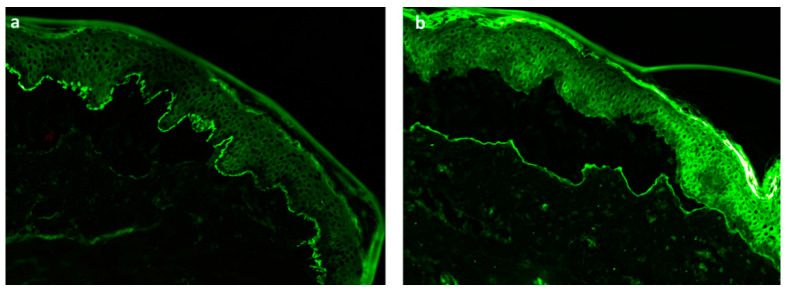
The salt-split skin technique is used to distinguish BP from EBA: (**a**) roof staining in bullous pemphigoid, (**b**) flor staining in epidermolysis bullosa acquisita. Magnification 200× (private photography) (online, in color; black and white, in print).

Despite everything, direct immunofluorescence examination does not distinguish BP from EBA, and the salt-split skin technique is necessary to distinguish EBA from BP (Figure 3). 

### 3.3. Dermis and Subcutaneous Tissues

The dermis is 0.5 to 5 mm thick, depending on the body site (thinnest on the eyelid, much thicker on the sole), and it is composed of extracellular matrix (ECM) components such as collagen, elastin, and glycosaminoglycans, with fibroblasts being the primary cell type. It is highly vascularized and is inhabited by dermal adipose cells, mast cells, and infiltrating leukocytes [39]. This skin layer could be divided into the papillary dermis (PD) and reticular dermis (RD) [40,41]. Collagen and elastic fibers are deposited by fibroblasts. In the dermis layer, we can find also histiocytes, antigen-presenting cells that phagocytose and degrade foreign substances and present antigens to T cells. These cells are responsible for the debridement of tissue through eating bacteria and dead cells, as well as tissue fragments, and foreign bodies. About 70% of the dry weight of the epidermis is made up of collagens, of which the predominant types are types I and III [30,41,42].

Directly below the dermis is where the subcutaneous layer is located, which connects the skin to the underlying fascia (fibrous tissue) of the bones and muscles. The thickness depends on the skin site, sex, and an individual’s nutritional habits. In the subcutis, the adipocytes are organized into lobules that are separated by septae of connective tissue. The adipose tissue has metabolic functions: it is responsible for the production of vitamin D and triglycerides [39].

## 4. Role of Keratin in Wound Healing

Keratins can be categorized as soft or hard keratins, depending on the tissue types in which they are expressed. The epithelial keratins (soft keratins) are found in the epidermis, oral mucosa, and other epithelial linings. They form intracellular intermediate filament networks which provide a scaffold for epithelial cells and tissues to withstand mechanical stress and maintain structural integrity. They are also involved in cell physiology processes, such as cell adhesion, migration, and differentiation [43]. Keratins accomplish these functions by interacting with a myriad of signaling proteins. Frequently, these interactions are mediated by elaborate post-translational modifications of keratins [44].

Apart from epithelial keratins, trichocytic keratins (or hard keratins) are found in epidermal appendages and are further classified into α-keratins (found in hair or wool) and β-keratins (found in avian feathers and reptilian tissues) [45]. Moreover, comparisons between the amino acids sequence of trichocytic α-keratins (e.g., sheep’s wool) and the epithelial cytokeratins showed that they are homologous in the α-helical rod domain, but they differ considerably in the head and tail domains. During development, the hair follicle germ-cells derive from the fetal epidermis. Therefore, it is important to establish whether the expression of epidermal-type cytokeratins is continued in the cells of the bulbar follicular base that are involved in hair formation [46]. It should be mentioned that hard keratins are a distinct sub-family of keratins having a higher sulfur content due to a larger proportion of cysteine residues in their primary sequences [43,45,46,47].

Keratins are responsible for the organized proliferation of the keratinocytes and maintaining their integrity in the epithelium. Keratins are the intermediate-filament-forming proteins expressed in epithelial cells and can be divided into two groups: keratins type I, also called acidic keratins (KRT9-KRT40); and keratins type II, called neutral-basic keratins (KRT1-KRT8) [48,49].

**Figure 4:**
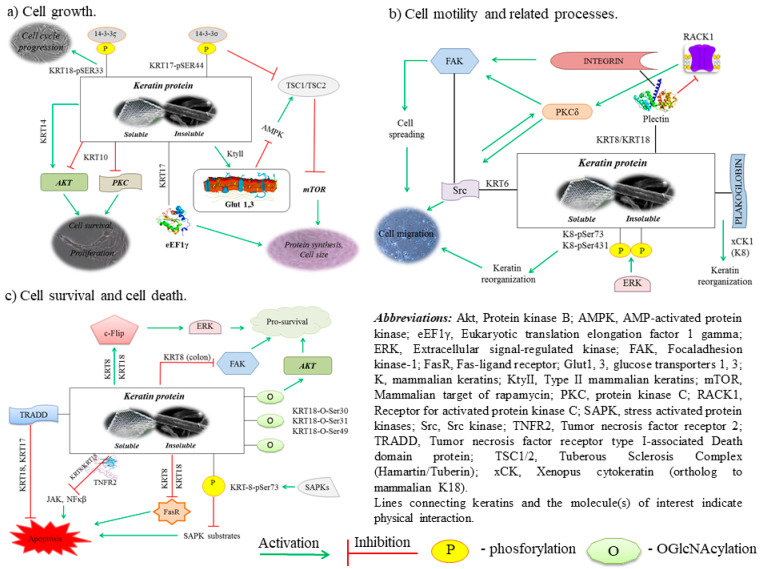
Keratin proteins regulate and participate in different cellular and physiological processes (online, in color; black and white, in print).

Both types of keratins are produced by activated keratinocytes. They are encoded by 54 evolutionarily conserved genes (28 types I, 26 types II) and regulated in a pairwise and tissue type-, differentiation-, and context-dependent manner [48,49]. In vivo keratins always exist as heteropolymers containing one type I and one type II keratin. The expression of different pairs of these keratin monomers varies from layer to layer, depending on the level of the dermis. For example, the biggest expression of KRT14-KRT5 occurs in basal layers of keratins, while a large amount of KRT10-KRT1 is present in suprabasal levels of the epidermis [50]. It should be mentioned that keratins are involved in intracellular signaling pathways, e.g., protection from stress, apoptosis, and wound healing (Figure 4) [44,49].

The most important keratins involved in wound healing are KRT16/KRT17-KRT6. During the first hours after wounding, KRT16/KRT17-KRT6 is being highly expressed, at the cost of KRT10-KRT1, which makes it easier to promote proliferation over differentiation [51].

Studies achieved using the null-mice, allowed researchers to discover the function of keratins in the wound healing process. Mice with KRT6a and KRT6b deletions had not shown any pathological symptoms at birth, but, after a few weeks, they died due to massive oral epithelium blisters, followed by poor nutrition [52]. Although, when KRT6a/KRT6b null skin was grafted into healthy mice, this fragment was more fragile to damage. Besides that, lysis of keratinocytes and swelling was noted. In an ex vivo KRT6a/KRT6b null skin fragment, there was a decreased concentration of KRT16, even despite normal KRT16 mRNA levels. Due to this fact, it is believed that KRT6 is needed to support the stability of KRT16, which in vivo creates KRT6/KRT16 heteropolymers [53]. On top of that, KRT6 directly interacts with SRC kinase, decreasing its activity, which weakens cell migration in wounded skin. Migrating cells lacking KRT6a/KRT6b proteins showed significantly decreased cell-cell adhesion, usually leaving gaps between cells. In contrast, healthy cells from the control group made organized structures after cell migration [53,54].

Keratin-17 (KRT-17) is responsible for keratinocyte proliferation. KRT-17 null keratinocytes are smaller, have decreased speed of protein translation associated with decreased mTOR/AKT signal, which has a direct impact on keratinocytes proliferation [55]. Another role of KRT-17 is to support the phosphorylation of STAT3 and the transportation of this protein to the cell nucleus. This increases translation of D1 cyclin that is necessary for the proper proliferation of keratinocytes [56]. 

In acute wounds, keratinocytes migrate from the wound margins, proliferating and releasing cytokines to initiate tissue response. However, impaired wound healing a special in diabetes can cause chronic wounds, such as ulcers, where keratinocytes appear to be unable to migrate, leaving this phase of wound healing incomplete [57].

It should be mentioned that some classes of keratin exhibit characteristic expression patterns in human tumors. Some of them, especially K5, K7, K8/K18, K19, and K20, have great importance in immunohistochemical diagnosis, particularly of unclear metastases and precise classification, as well as subtyping of carcinomas [58].

## 5. Biomedical Properties of Keratin

Many scientific groups around the world look for new therapies, regeneration methods, which are based on the use of natural-derived biomaterials that can support wound healing. Knowing the physicochemical and biological properties of different biomaterials, we can create a new material that can be used for biomedical applications, including wound dressing or drug delivery systems [59]. In this context, keratin biomaterials possess a unique set of properties, such as excellent biocompatibility, biodegradability, and bioactivity. They also possess a hydrophilic surface, which is absent in many synthetic polymers [60]. To appropriate understanding and use the following terms: biocompatibility, biodegradability, and bioactivity term, it is necessary to introduce basic definitions of them.

Biocompatibility can be defined as the ability of materials to interact with host cells without causing systematic and local cytotoxicity, mutagenesis, carcinogenicity, allergic reactions, or inflammation [61]. Several in vitro studies on different cell lines have been shown that keratin biomaterials are biocompatible and support cell growth [62,63,64]. Yamauchi et al. [65] proposed that keratin-coated substrate has shown better L929 fibroblasts attachment and growth compared to the ones coated with collagen. Park et al. [66] have also reported that keratin extracted from human hair is more effective in healing wounds due to the almost complete regeneration of epithelial cells. Sadeghi et al. [67] examined carboxymethyl cellulose (CMC)-human hair keratin hydrogel with controlled clindamycin in vitro on mouse fibroblasts. They showed that increasing the content of keratin in CMC hydrogel not only lowered water vapor transmission rate to a suitable range for wound healing but also improved the water stability of CMC hydrogel. The in vitro release study indicated that clindamycin was released slower in samples containing higher keratin. Higher keratin content led to better cellular attachment, proliferation, and spreading. Summarizing, the addition of keratin into the carboxymethyl cellulose improves their properties compared with pure CMC samples.

Another important parameter described biomaterials in the field of tissue engineering and regenerative medicine is bioactivity. Bioactivity is closely related to biocompatibility and can be broadly defined as “one which has been designed to induce specific biological activity” [68]. Biodegradability is an essential property for designing biomaterials used in tissue engineer and regenerative medicine. Despite this, a typical scaffold should have similar mechanical properties to the host tissue. Biodegradability can be defined as the ability of a material to break down into its simpler components after contact with the biological environment after implantation [69]. The in vivo biodegradation of keratin was explored by the different research groups. Peplow et al. [70] examined wool keratin bars after subcutaneously implanted into adult rats for 18 weeks. They found that this gradual degradation and quick loss of mechanical integrity showed that this form of keratin is more appropriated as a resorbable implant material to provide a scaffolding framework for cell migration and proliferation. In another study, Bochyńska-Czyż et al. [64] examined in vitro keratin scaffolds derived from rat fur. They showed that co-cultures of rat mesenchymal stem cells on keratin biomaterial confirm the formation of 3D cell culturing on keratin scaffolds and can be maintained in vitro for several weeks without morphological changes of cells. This observation confirms good biocompatibility, low immunogenicity, and slow biodegradation of keratin biomaterials.

Apart from this, keratin biomaterials derived from wool and hair have shown the capability to support cellular attachment as they possess cell-binding motifs, such as glutamic acid-aspartic acid-serine (EDS), arginine-glycine-aspartic acid (RGD), and leucine-aspartic acid-valine (LDV) binding residues [22,71]. The presence of LDV and EDS binding residues support cellular attachment, while RGD acts as a binding motif for proteins, such as fibronectin, fibrinogen, and osteopontin [22,72].

### Antibacterial Properties of Keratin Biomaterials

Antimicrobial efficacy is one of the most important requirements for modern scaffolds for wound care, tissue engineering, and biomedical applications as a whole [73]. It should be mentioned that keratin itself exhibits antimicrobial activity, although it is not outstanding compared to the activity of silver nanoparticles, it is significant. This may be attributed to some important functional groups present in keratin protein, especially in the peptide backbone, such as disulfide (-S-S), amino acid (-NH2), and carboxylic acid (-COOH). Amino acid analysis shows that keratin has a high content of cysteine residues (7–20% of all amino acid residues). Thus, it appears that the binding of the positively charged amino groups of keratin and the negatively charged bacterial cell wall causes keratin to inhibit bacterial growth [73]. 

Regardless of the source of keratin (hair, fur, or feathers), the materials exhibit antimicrobial properties which can be enhanced by the addition of antimicrobial substances or drugs (e.g., AgNP or mupirocin) [10,74]. Konop et al. [7,10] showed that insoluble fraction of keratin scaffolds alone or with the addition of silver nanoparticles exhibit antimicrobial properties against *S. aureus* and *E. coli* in vitro. Moreover, they do not observe the sign of bacterial infection in keratin-treated wounds in diabetic mice. In addition, another group showed that keratin biomaterial alone or mixed with antibacterial agents possess antimicrobial properties.

He et al. [75] showed that the AgNPs-embedded FK/PVA/PEO nanofibers exhibited antibacterial activities against both Gram-positive (*S. aureus*) and Gram-negative (*E. coli*) bacteria. A clearer and larger circular inhibition zone were observed against *E. coli* compared with *S. aureus*. In another study, Amajuoyi et al. [75] examined keratin/coenzyme Q10/polyvinyl alcohol nanofibrous scaffold containing mupirocin and showed that obtained biomaterial possess excellent antimicrobial activity against all clinical isolates strains of Staphylococcus aureus. Sundaram et al. [76] examined the antimicrobial properties of keratin nanoparticles made from chicken feathers. They showed that the zone of inhibition was higher for *S. aureus* compared with *E. coli* (11 mm vs. 9.5 mm), which indicates that the obtained keratin nanoparticles can be effective against infections caused by bacteria.

On the other hand, single reports suggest that bacteria presenting keratinolytic properties may grow more likely in the environment enriched in specific keratin proteins. Nasipuri et al. [11] showed that four bacterial strains (*S. rhizophila*, *X. retroflexus*, *M. oxydans*, and *P. amylolyticus*) isolated from solid can degrade the keratin. *X. retroflexus* was the best keratin degrader in terms of culture density and keratin degradation, as well as combined with the non-degraders *M. oxydans* and *P. amylolyticus*, and/or *S. rhizophila* that did degrade keratin but grew slowly, to investigate community degradation as compared to single species.

Summarizing, independently from keratin source, the obtained biomaterials possess antimicrobial properties, are safe, and can be considered as a new type of wound dressing a specially in the case of chronic, non-healing wounds burdened with a bacterial infection.

## 6. Effect of Keratin on Hyperpigmentation

There is a growing list of keratin-related genetic diseases that point to new functions for keratin. One of these functions is a role in skin pigmentation. Some of the key supporting evidence comes from studies of unconventional forms of epidermolysis bullosa simplex (EBS) and other disorders resulting from KRT5 or KRT14 mutations [77,78]. Uttam et al. [79] found out that a peculiar missense mutation, Pro24→Leu, in the head domain of KRT5, causes EBS with mottled pigmentation, in which small (2- to 5-mm wide) hypo- or hyperpigmented spots confer a mottled appearance to the skin. Moreover, Betz et al. [80] have linked Dowling-Degos disease (DDD), which is characterized by reticulate hyperpigmentation and dark hyperkeratotic papules in skin flexural regions, to KRT5 haploinsufficiency. Further, dermatopathia pigmentosa reticularis (DPR) and Naegeli-Franceschetti-Jadassohn syndrome (NFJS) are two related dominantly inherited conditions featuring reticulate or mottled hyperpigmentation of the skin that are caused by frameshift mutations, which are located in the head domain of KRT14 [81]. 

Evidence supporting a novel role for keratin proteins in regulating skin pigmentation also comes from genetic animal models, primarily in mice. It is associated with mutations within the genes for KRT1, KRT2, and KRT4 [77,78]. Fitch et al. [82] used random chemical mutagenesis to identify genes involved in fur color determination in mice. Of the genes identified, dominant missense alleles in KRT2e (T500P) [82] or KRT1 (S194P) [83]. A third mutation, N154S, was found in the KRT4 gene in a mouse line showing a lighter than normal fur color [84]. It should be highlighted that the KRT4–KRT13 keratin pair is predominantly expressed in the stratified epithelial lining of the oral mucosa and esophagus [85]. In humans, mutations in KRT4 or KRT13 cause oral white sponge naevus (WSN), a rare condition primarily affecting the oral mucosa [86].

Loan et al. [87] presented a cohort study in which wounds of 40 patients who suffered from superficial and partial-thickness burns were observed and rated from 1 (normal skin) to 10 (worst scar imaginable), depending on a change of skin pigmentation in the wound area after using keratin-based treatment. Twelve months after keratin-based treatment, the skin was normally pigmentated in 73% of all patients, 20% of them were given 2 or 3 points, and only 7% were given >3 points. However, since there was no control group in this study, and hyperpigmentation after partial-thickness burn is being described regardless of the treatment method [88], further studies should be executed to evaluate the impact of keratin-based treatment on hyperpigmentation.

To summarize, there are reports of keratin expression in melanocytes, the significance of which remains unclear [89]. In-depth studies of how keratin proteins may be regulating melanosome import, as well as melanin distribution, will help characterize this newly emerging role of cytoplasmic keratin intermediate filaments.

## 7. Biomedical Propertied of Keratin Biomaterials

During the last few years, a lot of effort has been devoted to the development of functional wound care products which, apart from antimicrobial protection, contain ingredients that promote wound healing [90]. In this context, keratin biomaterials increased scientists’ attention due to excellent biocompatibility and the possible preparation of different forms of a wound dressing. Keratin biomaterials can occur in the form of gel, hydrogel, foam, films, and scaffolds [11]. A different form of keratin has been studied for its application as promising biomaterials for wound healing, bone regeneration, hemostasis, and peripheral nerve repair. It should be mentioned that lots of studies describe the application of a soluble fraction of keratin as a wound dressing [3,91,92]. However, in the literature, we can find only a few studies that described the insoluble fractions of keratins in wound treatment [7,10,93]. Moreover, the basic wound dressing can be modified with antimicrobial compounds, such as silver nanoparticles (AgNP), antibiotics, or other substances, that can improve the recovery of injured tissues and prevent secondary infection [10,23]. 

Regardless of the form in which it occurs, the dressings are tested on different wound healing models in laboratory animals. Chronic, non-healing wounds are a serious problem for people suffering from diabetes. Therefore, different scientific groups examined potential keratin-based dressings in healthy and diabetic conditions that can occur in laboratory conditions. In addition, single experimental studies described the application of keratin gel as a wound dressing in a patient with bullous diseases. 

Lin et al. [22] examined keratin biomaterial extracted from human hair in vitro and in vivo. They showed that the obtained biomaterial is biodegradable, biocompatible, and supported human adipose stem cells (hASCs) adhesion, proliferation, and differentiation. In vivo investigation performed on ICR mice showed that keratin scaffolds with hASCs shortened healing time accelerated epithelialization and promoted wound remodeling in comparison with the keratin-scaffolds-only group, hASCs-only group, and control. On the whole, keratin scaffolds possess appropriate pore size and good cytocompatibility and can be further applied in the field of regenerative medicine.

In the other study, Kim et al. [94] analyzed the opportunity to use keratin hydrogel made of human hair in the full thickness mouse excisional wound splinting model. Twenty-one days after wounding, they observed that, in keratin-hydrogel-treated mice, skin was fully recovered without any sign of scab and wound contraction, while, on the control side, scabbing was still visible. Histopathological examination showed that the rates of re-epithelialization, remodeling, and repair in the keratin-based hydrogel-treated group were 2-fold higher than those of the negative control group, which were calculated as 50, 37.5, and 12.5% in the negative control group, and as 20, 50, and 30% in the keratin-treated group. Subsequent studies, carried out by Li et al. [95], examined the influence of keratin hydrogel with the addition of insulin in full-thickness skin wound healing, scar creation, and hemostasis in Sprague Dawley rats. Rats were separated into four groups: control, keratin-treated, insulin-treated, and insulin-keratin-treated group (Ins-K). In the Ins-K hydrogel-treated group, higher hemostatic abilities were noted, compared to the keratin group. They observed that, between all of the groups, the fastest wound closure appeared in the Ins-K-treated group in the early stage of regeneration (first 2 weeks, *p* < 0.05). Another important fact is that wounds treated with Ins-K hydrogel have a more regular arrangement of the tissue and present the lowest TGF-β levels, which caused the inhibition of the fibroblast cells hyperplasia, and prevented the wound from scarring. Summarizing, the obtained results established that keratin possesses the abilities of hemostasis, wound healing, and scar inhibition after insulin conjugation and can be used as a safe wound dressing. 

Poranki et al. [96] examined keratin-hydrogel prepared from human hair, on skin regeneration after chemical and thermal burns in mice and swine. The in vitro thermal injury studies showed that gamma-keratose-treated fibroblasts did not show decreased cell viability at 24 h, characteristic for other experimental groups (crude and gamma keratose). Gamma-keratose-treated fibroblasts were able to increase cell viability to 125%, at the same point of the experiment, where alpha keratose grew to 46%, and crude keratose grew to 65%. In vivo studies performed on mice and swine set out that wounds treated with keratose hydrogel showed significantly faster wound closure, with complete wound closure within 18 days compared to 24 days in chitosan and saline-treated wound. This study showed that the gamma fraction, composed essentially of degraded alpha keratin proteins, may facilitate cell rescue after thermal injury. Treatment of burns with this type of keratin protein may, therefore, represent a potential therapy in the treatment of burn wounds in the future.

Gao et al. [97] investigated the wound healing ability of the recombinant human hair keratin proteins (RKNP37 and RKNP81) and keratin nanoparticles (KNP) in a full-thickness wound model in SD rats. In vitro studies performed on HaCaT keratinocytes showed significantly increased cell proliferation and migration in a dose-dependent manner compared to control. RKNP37 and RKNP81 in the concentration of 1.000 mg/mL showed increased cell growth of 250% and 310%, respectively, compared to the concentration of 0.000mg/mL. They observed that the RKNP81 displayed a stronger cell proliferation ability than RKNP37. Open-excision full-thickness wounds were created on the rat’s dorsum and covered with 0.500 mg of RKNP37, RKNP81, or KNPs and fixed with Tegaderm film. A wound covered with Tegaderm film serves as a control. In vivo experiments showed that KNPs and the RKNPs significantly accelerate wound healing within 2 weeks, and the RKNPs displayed a stronger wound-healing ability than KNPs. In addition, wounds treated with RKNP81 revealed a stronger wound-healing effect compared to RKNP37. To summarize, the RKNP’s treatment of dermal wounds resulted in improved epithelialization, vascularization, collagen deposition, and remodeling. The in vivo biocompatibility test revealed no systemic toxicity. This biomaterial did not induce tissue inflammation nor immune responses that were biocompatible and biodegradable.

Since radiotherapy combined with surgery is a common therapy in malignant tumor therapy, Chen et. al. [98] examined hydrogels containing keratin extracted from the human hair, as a potential biomaterial useful in the treatment of irradiated full-thickness skin wounds. In vitro studies performed on HaCaT keratinocytes irradiated with 5 Gy of gamma radiation showed that, after 48 h, cell viability was significantly higher (*p* < 0.01) in the keratin irradiated (K + IR) group (~120%) compared to the non-keratin irradiated (IR) group (~60%). In vivo studies consisted of two stages: rats were exposed to 5 Gy of gamma radiation, and then a surgical procedure was performed. In keratin hydrogel-treated wounds, the faster repair was observed when compared to other groups. It has also been observed that irradiated keratin-treated wounds showed a regular reduction of wound area every 7 days until the full closure. It was further noted that irradiated wounds without hydrogel treatment were only marginally healed.

Konop et al. [8] examined insoluble keratin dressing named fur keratin-derived protein (FKDP) in a full-thickness skin wound model in healthy mice. They showed keratin biomaterial is not only toxic to the NIH/3T3 cell line but also enhances cell proliferation compared with the control (*p* < 0.05). In vivo studies have shown that keratin dressing is tissue biocompatible and accelerates epithelialization compared to the control side. It should be mentioned that FKDP-traded wounds healed faster on day 5 (*p* < 0.05) in comparison to control wounds. They also pointed out that, in FKDP-treated wounds, predominant macrophages and the epidermis were thicker. The presence of the mentioned cells creates a more favorable environment for healing. They also argued that keratin dressing favors the reconstruction of a more regular skin structure and assures a better cosmetic effect in terms of scar formation and appearance. 

Shanmugasundaram et al. [99] describe the application of nonwoven wound dressings made from chicken feather keratin (CFK-NW), keratin-sodium alginate (CFK-SA-NW), and keratin-chitosan (CFK-CS-NW) in a partial thickness skin wound in Wistar rats. Antimicrobial studies confirmed a positive antibacterial effect against *S. aureus*, *K. pneumoniae*, and *E. coli* bacteria. In vitro examination on L929 mouse fibroblast showed that all obtained keratin biomaterials are non-toxic and support cell viability. It was shown that wounds treated with CFK-CS-NW and CFK-SA-NW dressings heal faster compared to wounds treated with CFK-NW and untreated control.

Vakilian et al. [100] proposed a new type of keratin biomaterial derived from the shed skin of two different Omani snakes (Puff and Cat Snakes) as a wound dressing in full-thickness skin wound healing in Wistar rats. Rats were divided into 4 groups: group 1, positive control (PC) treated with Solcoseryl ointment, group 2 where wounds were covered with Puff snake shed skin (P), group 3 with wounds covered with Cat snake shed skin (C), and group 4 which served as a negative control, i.e., without any treatment. Animal studies showed that skin reconstruction was effectively improved under P shed skin treatment when compared with the negative and positive control (PC) and C-treated groups. Moreover, the authors suggest that P snake shed skin might heal acute wounds either directly or indirectly via the release of inflammatory cytokines and granulation tissue formation, resulting in reepithelization, neovascularization, and fibroblast expansion.

### Diabetic Condition 

Impaired wound healing is a medical problem, especially in patients with diabetes. Many scientists have looked for new therapeutic strategies in the field of chronic non-healing wounds. In this case, naturally derived biomaterials, such as keratin, are promising agents which can be used in the future as a wound dressing. 

Veerasubramanian et al. [101] tested hydrogel composed of konjac glucomannan (KGM), human hair proteins (KER), and an ethanolic extract of Avena sativa (OAT) as a potential biomaterial for diabetic wounds in rats. The in vitro examination showed that the obtained hydrogel is biocompatible, non-toxic for the NIH/3T3 cells, and possesses antimicrobial activity against *S. aureus* and *E. coli*. Wounds treated with hydrogels (KGM + KER + OAT) undergo faster re-epithelialization, and a thick epithelial layer and matured collagen were visible on day 12. Masson-trichome staining showed that the KGM + KER + OAT scaffold aids collagen synthesis and deposition, which leads to accelerated wound healing in diabetic rats. Complete wound closure was observed on day 16 in the test group treated with KGM + KER + OAT compared with KGM + KER dressings and the control, respectively (day 20 and 24 post-injury).

Ponrasu et al. [102] investigated hydrogel scaffolds composed of morin, psyllium, and keratin (PSH + KER + MOR) as a potential biomaterial that supports skin wound healing in diabetic Wistar rats. They noted that, cell viability was similar (around 82%) in all three groups: PSH + KER, PSH +KER + 0.5% MOR, and PSH + KER + 1% MOR after 48-h observation. Antimicrobial studies showed that MOR scaffolds had a bacteriostatic effect against *S. aureus*. On the other hand, PSH + KER hydrogels showed poor properties against gram-negative bacteria (*E. coli*). In vivo wound healing process in diabetic rats was observed in every 4-day interval. Animal studies showed that PSH + KER + MOR scaffold treatment significantly reduced the re-epithelialization time and enhanced the rate of wound contraction by accelerating collagen synthesis in diabetic rats compared to controls. 

Konop et al. [7] tried to determine the possible application of an insoluble fraction of fur-derived keratin biomaterial as a wound dressing in a full-thickness surgical skin wound model in diabetic mice. They showed that keratin dressing significantly (*p* < 0.05) accelerated healing during the first week after surgery compared to control wounds. The dressings were incorporated naturally into granulation and regenerating tissue without any visible signs of the inflammatory response, which was confirmed by clinical and histopathological analysis. Therefore, it can be considered a safe and efficient wound dressing. 

To improve antimicrobial properties, the obtained keratin dressing can be supplemented with various antimicrobial agents. Konop et al. [10] modified basic keratin dressing with silver nanoparticles and evaluated it in a full-thickness skin wound model in diabetic mice. Microbiologic results revealed that the insoluble FKDP-AgNP dressing, to some extent, contributed to inhibit growth of *E. coli* and *S. aureus.* In vitro assays showed that the FKDP-AgNP dressing did not inhibit fibroblast growth nor induce hemolysis and was biocompatible properties. FKDP-AgNP significantly accelerated wound closure and epithelization at days 5 and 8 (*p* < 0.05) when compared with control wounds. Histological examination of the inflammatory response documented that FKDP-AgNP-treated wounds contained predominantly macrophages, while their untreated variants showed mixed cell infiltrates rich in neutrophils. Wound inflammatory response based on macrophages favors tissue remodeling and healing. 

In a subsequent study, Konop et al. [93] modified keratin biomaterial (base dressing FKDP) with opioid peptide–casomorphin and examined in vitro and in vivo in diabetic mice model of full-thickness wound model. They showed that casomorphin slowly releases from the wound dressing. They pointed out that keratin-casomorphin dressing is biocompatible, non-toxic, and supports cell growth. In vivo experiments demonstrated that keratin-casomorphin dressing significantly (*p* < 0.05) accelerates the whole process of skin wound healing to its final stage. Wounds covered with keratin-casomorphin dressing underwent reepithelization faster, ending up with a thicker epidermis than control wounds. They showed that applied keratin dressing favored reconstruction of more regular skin structure and assured better cosmetic outcomes in terms of scar formation and appearance. Furthermore, obtained wound dressing created a favorable microenvironment and supports skin wound healing in diabetic mice. 

All studies performed by Konop et al. [7,8,10,93] showed the possibility of applying an insoluble fraction of keratin biomaterial as a wound dressing. Moreover, due to heterogeneous surfaces, it is possible to incorporate compounds with antimicrobial or analgesic properties. However, the authors should perform future studies that are needed to explain the molecular mechanism behind the fur-derived keratin effect during the multilayer wound healing process. Their findings may open the way for a new class of insoluble fur keratin dressings in chronic difficult-to-heal wounds treatment. 

All mentioned studies have shown that, regardless of the source of keratin (hair, fur, feather chicken, or shed snakeskin), the obtained biomaterial is tissue biocompatible and supports cell growth in vitro. Experimental studies in healthy and diabetic animals showed that keratin-treated wounds healed significantly faster compared to control wounds (Table 1) and underwent faster reepithelization with minimal scar formation. Moreover, examined dressings are tissue biocompatible and do not induce inflammatory reactions after implantation

## 8. Clinical Application of Keratin Biomaterial in Wound Healing

In recent years, the use of new keratin-based wound dressings represents a novel approach to wound management in clinical practice (see Table 2). It should be mentioned that all examined keratin dressings were made from sheep’s wool. Only a few studies described the application of a different form of keratin dressing in the treatment of chronic non-healing wounds or wounds observed in a patient with genetic disorders resulting from mutations in the genes encoding keratin [103]. Missense mutations in keratin 5 or keratin 14, highly expressed in the basal epidermis, cause the severe skin blistering disease epidermolysis bullosa (EB) in humans by rendering the keratin cytoskeleton sensitive to mechanical stress [103,104]. In addition, keratin-based dressing is a new therapeutic option for patients with autoimmune diseases, e.g., in different variants of epidermolysis bullosa [105,106].

Than et al. [107], in their research, presented three patients with recalcitrant, venous, mixed venous, and arterial leg ulcers treated with keratin dressing: Keraderm (Blacksburg, Virginia), which is a robust matrix dressing derived from freeze-dried keratin protein, and Kerafoam (Onset Dermatologics, Cumberland, RI, USA), which is an absorbent polyurethane foam dressing with a laminated keratin film. It should be mentioned that, in the past, these patients have appropriate standard treatment. They observed that, after the application of keratin dressing, venous ulcer completely healed after 30 weeks. However, this study has some limitations, and the authors could not exactly determine the mechanism of wound improvement in these cases. They only suggested that improved healing can be associated with the application of keratin dressing. Moreover, these dressings were found to be comfortable and easy to use by the patients and nurses.

In another study, Davidson et al. [108] attempted to determine if the experimental keratin dressing Keramatrix^®^ (Keraplast, San Antonio, TX, USA) accelerates epithelialization rates during healing of partial-thickness wounds, relative to a Standard Care dressing (Algisite (Smith and Nephew, London)). They examined it, on two groups of patients, before and after 50 years old (n = 26). They noticed that, in older patients (>50 years), wounds healed more slowly. However, they observed that there was significantly more epithelialization after 7 days in wound parts treated with the keratin dressing compared with standard care. On the other hand, they observed that, in the younger patients, epithelization was almost complete at 7 days (median epithelialization percentage, 80%). It should be mentioned that there was no significant difference in epithelization score between the keratin-treatment and control sides. This researcher suggested that the dressing may be clinically useful in similar situations where epithelialization may be delayed. 

Kirsner et al. [106] used a keratin-based wound dressing in an infant patient with ESB. They applied KeragelT^®^ (Keraplast, San Antonio, TX, USA) directly on the wound on the left hand and left foot and protected them with the same secondary dressings used as part of standard care. The right foot and hand healing continued with standard care and served as controls. Both dressings were changed 4 times per week. After 6 months of treatment, they observed faster-wound healing and decreased the number of blisters on the treated side (6 and 5 blister episodes on treated foot and hand, respectively, and 12 and 14 on control foot and hand, respectively) and decided to treat the control limbs. The authors hypothesized that keratin dressings enhance keratinocyte activity and accelerate epithelialization and wound closure. Moreover, keratin-based dressings improved quality of life and reduced the cost of care.

Than et al. [105] also examined Keragel^®^ (Keraplast Technologies, LLC, San Antonio, TX, USA) as a wound dressing in an 11-year-old patient with diagnosed recessive dystrophic epidermolysis bullosa. The back of the neck was treated daily with 20 g of Keragel^®^. After 3 months of treatment, a significant improvement was observed in the skin: the skin was much more robust, the incidence of blistering was much lower, and the wound had effectively healed. They observed a progressive, sustained improvement continued for the 12-month duration of the study. However, this study possesses some limitations: for example, lack of control side and the exact mechanism for the improvement is not fully understood and needs further study. 

In addition, Dayner et al. [109] examined keratin gel (KeragelT^®^, Keraplast Technologies, USA) in the management of wounds in 10 patients with different variants of epidermolysis bullosa (EB). The keratin gel was found tolerable and acceptable by 8/10 patients; however, itching was noted in two patients with Junctional EB, generalized as severe, who discontinued use. The other patients reported reducing itching. Of the remaining eight, six continued long-term use of gel until the wound healed, and it was used for subsequent wounds that developed, as well. The localized epidermolysis bullosa simplex (EBS) patients discontinued use of the gel after no response was seen following four weeks of treatment. In conclusion, in the six patients in whom the keratin gel was effective, the healing times were faster, and healed skin was more resilient. The obtained results from this study are consistent with previous reports about healing improvement in patients with different variant bullosa diseases. 

Another study, performed by Batzer et al. [110], describes the use of keratin-based wound products on refractory wounds. In this study, 45 chronic wounds of mixed etiologies presenting in 31 patients were treated with a solid keratin matrix (Keramatrix^®^) and a liquid keratin gel (Keragel^®^) wound dressing (Keraplast Technologies, San Antonio, TX, USA). They showed that topical keratin products resulted in improved healing in 82% of wounds that either healed (64%) or reduced in size by >50% during treatment. They also suggested that, in case of a wound infection, keratin treatment should be briefly interrupted, and antibacterial treatment should be started as soon as possible, and then application of keratin products should be restarted. 

Paulsen and Bygum [111] studied keratin gel (Keragel^®^, Keraplast Technologies, LLC, Christchurch, New Zealand) as an adjuvant in the treatment of recalcitrant pyoderma gangrenosum ulcers in a 62-year-old woman. Earlier, the standard treatment (e.g., corticosteroids, cyclosporine, and infliximab) was unsuccessful. Treatment was initiated with the keratin gel as an alternative to intensifying immune suppression and was effective. In the next visit, after 9 days of treatment with the gel, the ulcers were diminished in size, and the systemic treatment with infliximab, corticosteroids, and methotrexate in tapering doses was continued, along with Keragel^®^ as a topical treatment. In November 2015, after seven months of treatment, the ulcers were healed. Keragel^®^ seems to be a safe and effective topical adjuvant in ulcers of immunological origin and is especially useful in promoting the last steps in the healing process. However, more controlled studies are needed to establish the usefulness of the keratin gel in the treatment of pyoderma gangrenosum ulcers.

## 9. Concluding Remarks and Future Challenges

Traditional wound dressings, such as natural or synthetic cotton wool, lint, bandages, gauzes, etc., with varying grades of absorbance capacity, have been used for wound management. From the general point of view, traditional dressings are indicated for clean and dry wounds with mild exudate levels or used as secondary dressings. Modern medicine is moving away from dry wound therapy. The traditional wound dressings are replaced by modern wound dressing with more advanced formulations to provide a moist environment to the wound and enhance healing.

Different types of keratin-based biomaterials have been developed over the last decade for biomedical applications, such as sponges, hydrogels, wound patches, films, and fibers. The different biomaterials could be applied, depending on the type of wound. The main function of this dressing is to create a moist environment and stimulate wound healing. Tissue engineering methods allow surface modification of biomaterials with antimicrobial or anti-inflammatory agents, to enhance tissue recovery. Another advantage of this material is that it is of natural origin, and some keratins are involved in skin morphogenesis. Keratin biomaterials are biocompatible, biodegradable, and support cell growth. As a part of the epithelial cytoskeleton, keratins are important for the mechanical stability and the integrity of epithelial cells and tissues. Regardless of the form of the keratin dressing, these biomaterials stimulate epithelization and possess hemostatic properties. Wounds treated by keratin dressing are characterized by a more ordered tissue structure, they are covered with the epidermis layer more quickly (the epidermis is thicker compared to control wounds), and they heal faster overall. The opportunity for modification of basic dressing with bioactive substances or antibiotics, which are slowly released from it is another advantage. 

Although such promising results of experimental studies, we still do not know what molecular mechanisms are involved in the healing process. Some researchers suggested that the pathway of Akt-mTOR kinases participates in this process. Despite the promising results reported of the applications of these biomaterials, only a few of these progressed to clinical trials. However, these studies described single cases where keratin treatment was used. Multicenter studies of keratin dressings on a larger population of patients with wounds of various etiology are necessary to confirm that keratin dressing improves skin wound healing.

## Figures and Tables

**Table 1:** Summarizing of application of different keratin dressing in an experimental wound model.

Author	Source of Keratin	Type of Keratin Wound Dressing	Healing Rate (*p*-Value)	Wound Status	
				Control Wound	Dressed Wound
Experimental Studies					
Lin et al. [22]	Human hair	Keratin scaffold seeded with hASCs	*p* < 0.05.	Injected with 100 μL of PBS + semiocclusive adhesive dressing	Keratin scaffold seeded with hASCs + semiocclusive adhesive dressing
Kim et al. [94]	Human hair	Keratin-based hydrogel	*p* < 0.05.	PBS	Keratin-based hydrogels (100 μL)
Li et al. [95]	Human hair	Keratin hydrogel conjugated insulin	*p* < 0.05.	Untreated	Treated with keratin, insulin, and the Ins-K hydrogels
Poranki et al. [96]	Human hair	Keratin-hydrogel	Mice chemical burns model: at days 4 through 16. *p* < 0.05. Swine thermal burn model: at days 3, 6, and 12. *p* < 0.05.	Saline (occlusive dressing) and chitosan hydrogel	Keratin-hydrogel
Gao et al. [97]	Human hair	Recombinant human hair keratin proteins (RKNP37 and RKNP81) and keratin nanoparticles (KNP)	*p* < 0.05 on day 7, 14.	Tegaderm film	0.500 mg of RKNP37, RKNP81, or KNPs and fixed with Tegaderm film
Chen et. al. [98]	Human hair	Keratin hydrogel	*p* < 0.01 (Keratin and irradiated wound versus exposed and irradiated wound), *p* < 0.05 (Keratin and irradiated wound versus non-keratin non-irradiated wound).	Wounds exposed, one exposed and irradiated	Keratin hydrogel
Konop et al. [8]	Mice fur	Keratin scaffolds (FKDP)	*p* < 0.05.	No dressing	Keratin scaffolds
Shanmugasundaram et al. [99]	Chicken feather	Chicken feather keratin (CFK-NW), keratin-sodium alginate (CFK-SA-NW), and keratin-chitosan (CFK-CS-NW)	No available statistical analysis.	Nonwoven fabric	Chicken feather keratin (CFK-NW), keratin-sodium alginate (CFK-SA-NW), and keratin-chitosan (CFK-CS-NW)
Vakilian et al. [100]	Sneak shed skin (Puff and Cat Snakes)	Puff snake shed skin (P) Cat snake shed skin (C)	*p* < 0.05.	No dressing (negative control) Solcoseryl ointment (positive control)	Puff snake shed skin (P) Cat snake shed skin (C)
Veerasubramanian et al. [101]	Human hair	Konjac glucomannan-keratin hydrogel scaffold loaded with Avena sativa extracts	*p* < 0.05.	Group I—control, rats dressed in non-medicated cotton gauze	Group II–rats dressed with KGM + KER scaffolds; and Group III–rats dressed with KGM + KER + OAT scaffolds
Ponrasu et al. [102]	Human hair	Keratin hydrogel (KER) supplemented with Psyllium seed husk (PSH) or Morin (MOR)	*p* < 0.05.	Group I—rats dressed in cotton gauze	Group II–rats dressed with PSH + KER scaffolds; group III–rats dressed with PSH + KER + 0.50% MOR scaffolds; group IV–rats dressed with PSH + KER + 1% MOR scaffolds
Konop et al. [7]	Mice fur	Keratin scaffolds (FKDP)	*p* < 0.05.	No dressing	Keratin scaffolds (FKDP)
Konop et al. [10]	Mice fur	Keratin scaffolds (FKDP)	*p* < 0.05.	No dressing	Keratin scaffolds + AgNP (FKDP-AgNP)
Konop et al. [93]	Mice fur	Keratin scaffolds (FKDP)	*p* < 0.05.	No dressing	Keratin scaffolds + 0.1% Casomorphin

**Table 2:** Clinical application of keratin biomaterial as a wound dressing.

Author	Source of Keratin	Type of Keratin Wound Dressing	Healing Rate (*p*-Value)	Wound Status	
				Control Wound	Dressed Wound
Than et al. [105]	Sheep’s wool	Keragel^®^—keratin-based hydrogel	No data	Lack of control site	Keragel^®^
Kirsner et al. [106]	Sheep’s wool	KeragelT^®^—keratin-enriched gel	No data (healing reduced from 14 to 7 days)	Saline cleansing, soft silicone-based, nonadherent primary dressing, also absorbent foam dressing for the feet, a tubular gauze bandage wrap for hands	KeragelT^®^
Than et al. [107]		Robust matrix dressing derived from freeze-dried keratin protein	No data (venous ulcer completely healed after 30 weeks)	Lack of control site	Keraderm (Blacksburg, Virginia)
Davidson et al. [108]	Sheep’s wool	Keramatrix—absorbable matrix rich in keratin protein	No data	Alginate dressing	Keramatrix
Dayner et al. [109]	Sheep’s wool	KeragelT^®^—keratin-enriched gel	No data	Lack of control site	KeragelT^®^
Batzer et al. [110]	Sheep’s wool	Keramatrix, Keragel^®^	No data	Lack of control site	Keragel^®^, Keramatrix
Paulsen and Bygum [111]	Sheep’s wool	Keragel^®^	No data	Lack of control site	Keragel^®^

## Data Availability

Not applicable.
